# Chemoprophylaxis of precancerous lesions in patients who are at a high risk of developing colorectal cancer (Review)

**DOI:** 10.3892/mi.2024.149

**Published:** 2024-03-27

**Authors:** Nonna E. Ogurchenok, Konstantin D. Khalin, Igor S. Bryukhovetskiy

**Affiliations:** 1 Far Eastern Federal University, School of Medicine and Life Sciences, FEFU Medical Center, Russky Island, 690091 Vladivostok, Russian Federation; 2 Primorskiy Regional Clinical Hospital N1, Medical Center, Russky Island, 690091 Vladivostok, Russian Federation; 3 Far Eastern Federal University, Medical Center, Russky Island, 690091 Vladivostok, Russian Federation

**Keywords:** colorectal cancer, cancer stem cells, inflammatory bowel disease, biological therapy

## Abstract

The diagnostics of colorectal cancer (CRC) and precancerous lesions in the colon is one of the most urgent matters to be considered for the modern protocols of complex examination, recommended for use from the age of 45 years, and including both instrumental and laboratory methods of research: Colonoscopy, CT colonography, flexible sigmoidoscopy, fecal occult blood test, fecal immunohistochemistry test and stool DNA test Nevertheless, the removal of those precancerous lesions does not solve the issue, and, apart from the regular endoscopic monitoring of patients who are at a high risk of developing CRC, the pharmacological treatment of certain key pathogenic mechanisms leading to the development of CRC is required. The present review to discusses the function of β-catenin in the transformation of precancerous colorectal lesions into CRC, when collaborating with PI3K/AKT/mTOR signaling pathway and other mechanisms. The existing methods for the early diagnostics and prevention of discovered anomalies are described and categorized. The analysis of the approaches to chemoprophylaxis of CRC, depending on the results of endoscopic, morphological and molecular-genetic tests, is presented.

## 1. Introduction

As a lethal disease, cancer poses a challenge to the entire human race. Despite the progress made in modern medicine, cancer continues to be a leading cause of mortality worldwide. Moreover, the number of oncological diseases exhibits a steady growth rate due to poor ecology worldwide, particularly when considering the effect of microplastics on the epithelial barrier of mucous membranes (1), as well as the negative impact of agricultural pesticides (2), the nitrate poisoning of air (3)and drinking water (4), the consumption of red meat (5), and residing in proximity to industrial facilities (6).

Research is focused on socially important forms of cancer, including lung cancer, breast cancer and colorectal cancer (CRC), which is one of the most prevalent oncological diseases (7). Colonoscopy (8) with a subsequent morphological analysis of colon biopsy samples does not always suffice for the effective prevention of CRC (9-11). Additional pathomorphological tests on colon biopsy samples, involving molecular medicine methods, can significantly enhance the scope of diagnostics. However, the removal of precancerous lesions only does not solve the issue of effectively preventing CRC in high-risk patients. One of the possible solutions could be the development of pharmacological treatment protocols to regulate molecular mechanisms, responsible for the regeneration of cellular elements of the colon mucous membrane. In this context, special focus could be placed on decreasing β-catenin level that is one of the most critical indicators of the functional activity of neoplastic cells.

The present review discusses the methods of the pharmacological regulation of β-catenin in patients with precancerous colorectal lesions who are at a high risk of developing CRC.

## 2. Current state of affairs

Among the socially significant types of cancer, CRC stands out. At 60-70 years of age, the risk of developing the disease is highest and increases as one ages (12). The risk factors include hereditary genetic syndromes (Table I), communicable and non-communicable diseases, lifestyle risk factors (Table II) and inflammatory bowel disease (IBD).

A family history of CRC is one of the key risk factors, as well as the presence of syndromes, such as familial adenomatous polyposis (FAP), mismatch repair (MMR) gene mutations, Lynch, Muir-Torre, Peutz-Jeghers, Cowden, juvenile polyposis with autosomal dominant type of inheritance, serrated polyposis syndrome and Cronkhite-Canada syndrome with an unknown type of inheritance (Table I).

The results of multiple studies have demonstrated that patients with a positive family history have a lower risk of mortality due to CRC. A possible explanation for the link between a family history of CRC and improved survival rates is that those with a cancer-related family may make more conscious lifestyle choices, such as quitting smoking and increasing their physical activity. Another contributor could be more frequent and thorough screening that allows for the discovery of cancer at the early stages and, therefore, improves the chances of survival. Genetic differences between patients with CRC and those with no family history may be one more explanation for the existing contrast in survival rates. Studies have shown that patients with CRC and a positive family history are more likely to have heightened levels of microsatellite instability, which may lead to improved survival rates. Determining the influence, that family history has on the prognosis of CRC, is not an easy feat, since both genetic and environmental factors are to be considered; therefore, further research is required (13).

Among individuals aged 20-49 years, the incidence of CRC has increased in nine countries (Germany, USA, Australia, Canada, New Zealand, Great Britain, Denmark, Slovenia and Sweden), while only three countries have exhibited a decrease in its incidence (Italy, Austria and Lithuania) (14), and more than half of patients do not have the disease in their family history (15).

There is a consistent rate of morbidity among the elderly in North America, Europe and Oceania. A growing trend of the prevalence of CRC has been observed among young individuals with a high income in nine different countries on three different continents (14).

At the same time, the frequency of right-sided CRC makes it objectively more difficult to make a diagnosis during the endoscopic examination (16), as is the case with late-developing symptoms of colon obstruction due to a larger diameter of the right portion of the colon (16).

Colonoscopy is a gold standard for the diagnosis of colorectal neoplasms (16). Precancerous lesions are frequently discovered in patients aged ≥50 years during colorectal screening. The overwhelming majority of cases exhibit no symptoms, apart from occasional stomach aches and other signs of dyspepsia (17).

The diagnosis of precancerous lesions during colonoscopy is relatively easy; however, neoplasms in the proximal regions of the colon are poorly visible in a routine endoscopy (16) due to the larger diameter of the right-sided segments of the colon. This results in the later manifestation of intestinal obstruction and other CRC symptoms (16). The removal of such precancerous lesions is a crucial step in preventing CRC.

## 3. Clinical and morphological features of precancerous lesions in the colon

The European Society of Gastrointestinal Endoscopy recommends the cold snare method for the removal of polyps ≤9 mm in size. Diathermocoagulation together with mucous membrane lifting are used to remove sessile polyps ≤20 mm in size. If a polyp is pedunculated and >20 mm in size, those with a large head or a stem with >10 mm in diameter should be treated with a combination of diathermocoagulation and adrenaline injection into the stem or with one of the preventive mechanical techniques of hemostasis. However, larger size, visual signs of invasion and diagnostic extent of endoscopic ultrasonography are not always conclusive enough to select the only right method of polyp elimination (10); thus, an urgent morphological analysis may be required.

**Figure 1:**
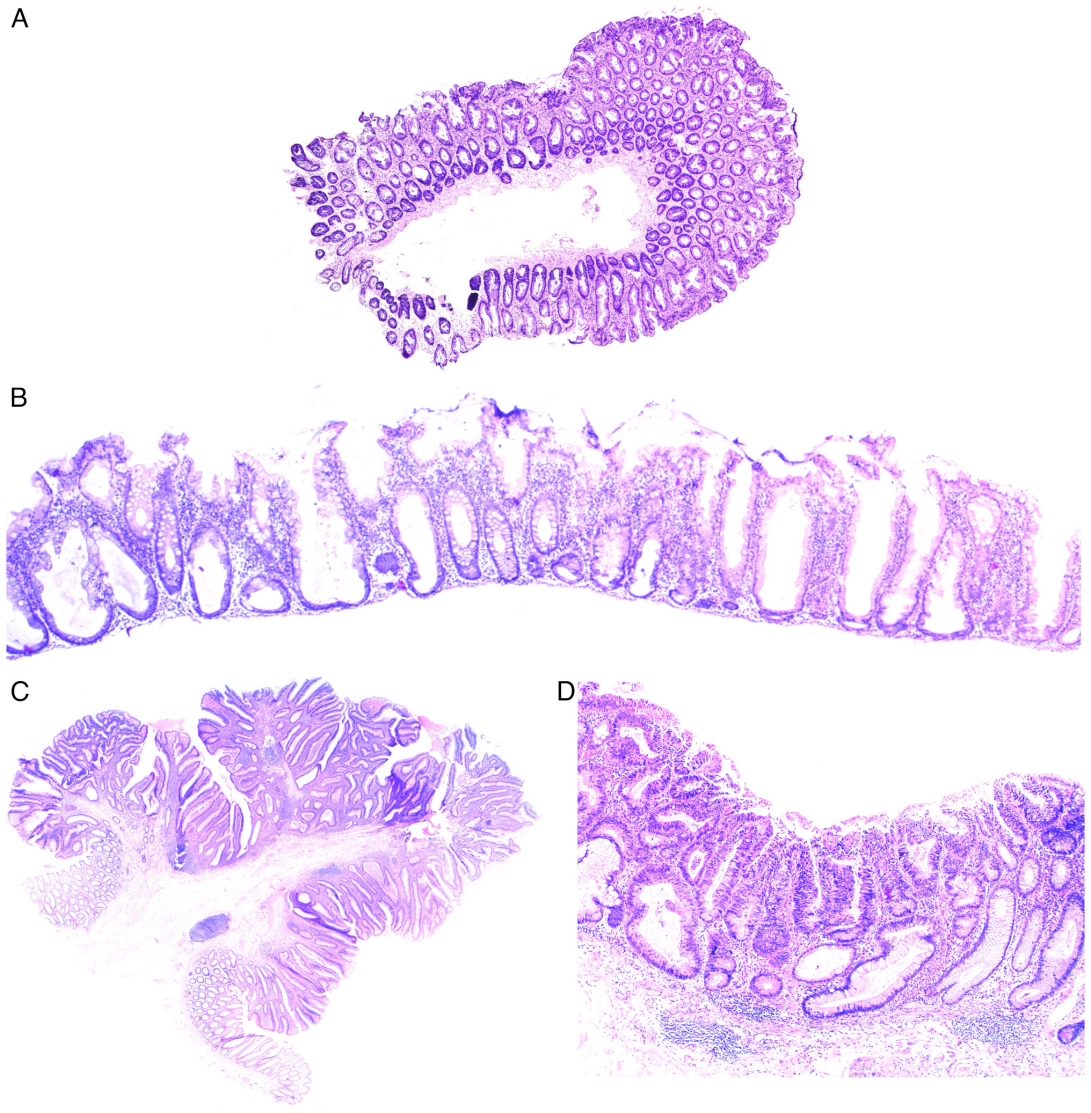
(A) A hyperplastic polyp, represented by a neoplasm with a serrated histoarchitecture, which develops as a result of impaired proliferation of the epithelium of the base of the crypts, which leads to the migration of proliferating cells into the apical parts of the intestinal crypts and the development of a serrated pattern. The tissue was obtained from a female patient, 70 years of age. The excision of the colon tumor was performed on May 22, 2023. A section of the mucous membrane of the colon measuring ~5.0 mm was prepared with further standard histological examination of the biopsy specimen and embedding in paraffin. Cutting was carried out using a microtome, with a section thickness of 4 µm. Standard hematoxylin and eosin staining was then performed. The magnification of the microscope cannot be reliably determined, since a series of images were taken with a magnification of x10 and subsequent stitching of the microphotographs. (B) Sissle serrated adenoma, represented by a neoplasm with basal localization of proliferation zones in the intestinal crypts and horizontal displacement of the proliferative zone, as well as the presence of dysplasia. The tissue was obtained from a female patient, 52 years of age. The excision of the colon tumor was performed on February 27, 2023. A flat section of the colon mucosa with a diameter of ~1.7 mm, with serial sections was prepared, with further standard histological processing of the biopsy specimen, and embedding in paraffin. Cutting was carried out using a microtome, with a section thickness of 4 µm. Standard hematoxylin and eosin staining was then performed. The magnification of the microscope cannot be reliably determined, since a series of images were taken with a magnification of x10 and subsequent stitching of the microphotographs. (C) Tubular adenoma, represented by a neoplasm with the presence of a classic tubular pattern with dysplastic changes, elongation, an increase in the number of tubules and the development of finger-like outgrowths. The tissue was obtained from a female patient, 49 years of age. The excision of the colon tumor was performed on June 1, 2023. The image depicts an exophytic neoplasm of the colon mucosa, pedunculated, 1.4 and 1.0 cm in size, with serial sections; further standard histological examination of the biopsy specimen and embedding in paraffin were performed. The section was cut was carried using a microtome, with a section thickness of 4 µm. Standard hematoxylin and eosin staining was performed. The magnification of the microscope cannot be reliably determined, since a series of images were taken with a magnification of x10 and subsequent stitching of the microphotographs. (D) Dysplasia in inflammatory bowel disease is characterized by a peculiar arrangement of dysplastic epithelial cells occupying the entire crypt. The tissue was obtained from a male patient, 66 years of age, with an established diagnosis of inflammatory bowel disease, namely ulcerative colitis. A colon biopsy was performed on November 30, 2022. A biopsy of the colon mucosa, 0.5 mm in size, with further standard histological examination of the biopsy and embedding in paraffin were performed. Cutting was then carried out using a microtome, with a section thickness of 4 µm. Standard hematoxylin and eosin staining was performed. The image is presented as x10 magnification with stitching of the microphotographs.

The World Health Organization (2019) (18) distinguishes CRC with serrated histoarchitecture, tubular and tubulovillous adenomas, focal intraepithelial neoplasia with underlying chronic inflammation (Table III). Moreover, as demonstrated in clinical practice, precancerous colorectal lesions often have mixed histological patterns with both serrated and tubular components, and there are no data either on what type of adenoma they should be classified as, or their molecular nature. Precancerous lesions with a serrated architecture appear due to the impaired proliferation in the cambium layer of intestinal epithelium in the crypt base, resulting in relocation of proliferating cells into the apical sections and development of serrated pattern (Fig. 1A) due to the competition for a place on a basal lamina (18).

The localization of proliferation zones in basal sections of colonic crypt without dysplasia indicators is typical for hyperplastic polyp, in contrast to which serrated adenoma is characterized by the horizontal displacement of a proliferative zone along its own muscularis mucosae, spread of serrated pattern into the crypt base, dilatation of its basal parts, asymmetric proliferation with dysplasia signs, formation of slit-like serrated structures and ectopic crypts similar to intussusception (Fig. 1B) (18).

The morphology of tubular adenomas shows the presence of glandular crypts, having mostly a typical architecture, exhibiting elongation, and increase in their number. The epithelium has enlarged hyperchromatic nuclei with varying degrees of stratification, spindle formation and loss of polarity, as well as a further overall decrease in the number of goblet cells. The specific feature of tubulovillous adenoma is the presence of finger-like projections, appearing as a result of crypt elongation (Fig. 1C).

A separate subtype of precancerous colorectal lesions is intraepithelial neoplasia in the case of IBD that creates additional complications for the differential diagnosis of serrated and conventional adenomas that are not IBD-related. The primary pathomorphological variation between IBD dysplasia and other precancerous lesions is considered to be the unique localization of dysplastic cells, which typically occurs in the upper crypts during sporadic adenomas and in the entire crypts during colitis (18,19) (Fig. 1D). In reality, morphologists usually cannot assess the exact localization of dysplastic cells due to the nature of tangential section, the incorrect placement of the sample in paraffin-embedded blocks, etc. One of the ways to solve this issue is using molecular genetic diagnostics.

## 4. Molecular markers of CRC

Reduced DNA methylation and associated epigenetic disorders of gene expression (20,21) play a particularly critical pathophysiological role in the development of CRC. The disruption of DNA methylation is caused by mutations in DNA methyltransferase (*DNMT*) genes responsible for the genome-wide methylation patterns (22); in particular, the methylation status of the promoter regions of the methylguanine-DNA-methyltransferase gene (23,24) he disruption of which is predominantly associated with mutations in the *KRAS* and *BRAF* genes (25), which are found in 22% of hyperplastic polyps, 25% of sessile serrated adenomas, and 50% of serrated adenocarcinomas (24).

The proteins, bone morphogenetic protein 3, N-myc downstream-regulated gene 4, annexin A10 (25-27), Runt-related transcription factor 3, suppressor of cytokine signaling 1, neurogenin 1, calcium channel, voltage-dependent, T type, alpha 1G subunit, insulin-like growth factor 2, p16, human mutL homolog 1 and MSX2-interacting nuclear target protein (28-30) have been described as markers of impaired genomic methylation status for CRC. The impairment of the DNA methylation status is typical for right-sided location of CRC, female patients, individuals of an advanced age (31), and high levels of genomic instability (32-34).

Genomic instability is a natural result of mutation accumulation and a driving force of the neoplastic process. A central role in the development of this phenomenon belongs to epigenetic failures in the expression of MMR genes, which function as a system of correcting improperly paired nucleotides, deletions or inclusions of incorrect bases, occurring during DNA replication. The proliferation of cells with an unstable genome is accompanied by the development of aneuploidy, which is observed in 65-85% of sporadic colorectal tumors (35-38), and is often accompanied by mutations in the adenomatous polyposis coli (*APC*), tumor protein p53 (*TP53*), catenin beta-1 and phosphatidylinositol-4,5-bisphosphate 3-kinase catalytic subunit alpha genes, in 20% of cases (39). *APC* gene mutation can initiate both tubulovillous (40-42) and serrated adenomas (43,44).

Among the first markers to exhibit an increase in expression, are the non-coding RNAs, CCAT1 and HOTAIR, in the plasma of patients with CRC; their expression was found to be significantly higher in patients with CRC than in those of the healthy controls. Other circulating IncRNAs that have been described as potential biomarkers for CRC detection include LOC285194, RP11-462C24.1 and Nbla12061, 91H, PVT-1 and MEG3, NEAT1 and NEAT2(45).

The serum levels of insulin-like growth factor binding protein-2 are elevated in patients with colon cancer, which is associated with neoplastic changes in the colon and carcinoembryonic antigen concentrations (45), as well as the overactivation of pyruvate kinase M2.

Dbf4-dependent kinase genes, which inhibit the Wnt-signaling pathway, are epigenetically silenced in CRC cells due to promoter hypermethylation. The activation of DKKS through small interfering RNA promotes the growth and invasion of cancer cells *in vitro* (45).

When precancerous lesions transform into CRC, a number of molecular mechanisms are activated, resulting in an increased level of β-catenin in pathologically altered cells. The Wnt signaling pathway plays a crucial role in this process.

## 5. Role of β-catenin in the transformation of precancerous lesions into CRC

β-catenin plays a crucial role in the Wnt signaling pathway, which regulates the balance between the symmetric and asymmetric division of both normal stem cells and cancer stem cells. β-catenin binds to the multiprotein destruction complex, which includes APC, Axin, casein kinase 1 and glycogen synthase kinase (GSK)-3, without the presence of a Wnt ligand. The secondary consequence is that GSK-3 phosphorylates β-catenin, leading to its ultimate degradation in the proteasome.

Wnt-ligands uses the transmembrane receptors, Frizzled and low-density lipoprotein receptor-related protein 4/5, to affect the cells. This complex activates the cytoplasmic protein Dishevelled, stops β-catenin degradation and inactivates GSK-3β, leading to accumulation of β-catenin in the cytoplasm and creation of conditions, allowing β-catenin to enter the nucleus, activate T-cell factor/lymphoid enhancer-binding factor and triggers the expression of *C-MYC*, cyclin-dependent kinase 4, WNT1-inducible signaling pathway protein-1, peroxisome proliferator-activated receptor γ (*PPAR-γ*) and other genes.

As an alternative mechanism for increasing β-catenin synthesis in precancerous lesions cells, both the direct activation of the phosphatidylinositol-3-kinase (PI3K)/protein kinase B (AKT)/mammalian target of rapamycin (mTOR) signaling pathway, which is the predominant signaling pathway that inhibits apoptosis, and the direct activation of intracellular AKT have been described.

PI3Ks are intracellular lipid kinases involved in the regulation of cell proliferation, differentiation and survival, that promote cell growth by inhibiting apoptosis in colorectal cancer cells and influences the effectiveness of chemotherapeutic agents (46).

**Figure 2:**
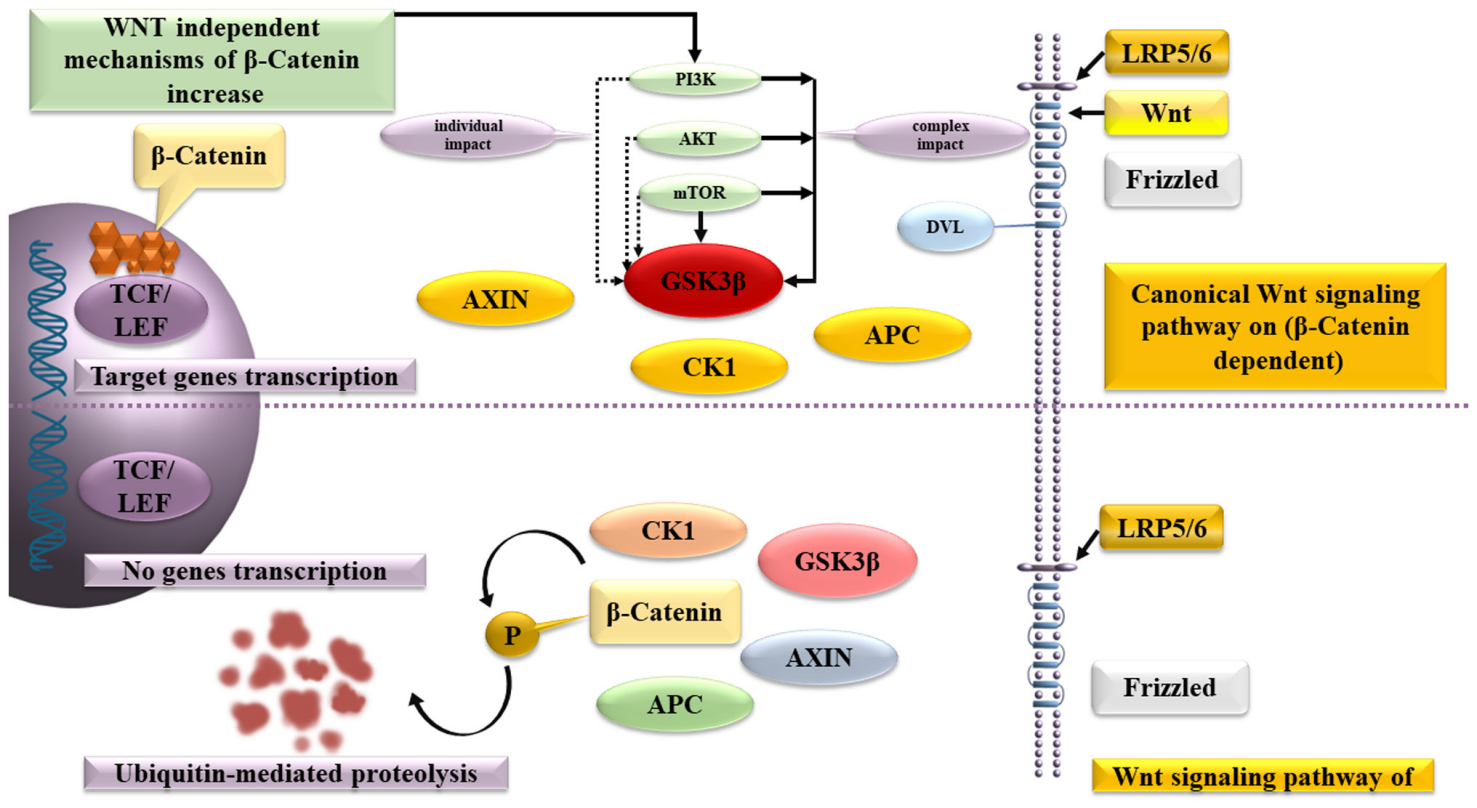
Pathogenesis of β-catenin accumulation in the canonical and non-canonical WNT signaling pathway, as well as alternative mechanisms of nuclear β-catenin accumulation in the nucleus with a predominant effect on GSK-3β. GSK-3, glycogen synthase kinase-3; TCF/LEF, T-cell factor/lymphoid enhancer factor; AXIN 1/2, AXIS inhibition protein; CK1, casein kinase 1; APС; PI3K, phosphatidylinositol-3-kinase; AKT, protein kinase B; mTOR, mammalian target of rapamycin; DVL, Dishevelled; LRP5/6, low-density lipoprotein receptor-related protein.

*AKT* (*AKT* proto-oncogene), which plays a key role in several types of cell death, as well as in the destruction of extracellular signaling molecules, oxidation, osmotic stress and ischemic shock, phosphorylates GSK-3Β and increases the content of β-catenin in the cancer stem cells. In turn, mTOR forms two multiprotein complexes: mTorc1 and mTorc2, which are capable of antagonistically regulating each other's activity, while the first reduces and the second increases the content of β-catenin (Fig. 2).

A higher β-catenin level activates telomerases (tert), causing their elongation, the immortalization of pathologically altered cells and the increased production of transforming growth factor β (TGFβ), that uses the SMAD and DAXX, death-associated protein 6 signaling pathways to activate the PI3K/AKT/mTOR axis. This results in the accumulation of β-catenin (47) and the triggering of epithelial-mesenchymal transition (EMT) in precancerous lesion cells (48-50), accompanied by activation of Snail, Twist, Slug, zinc finger E-box binding homeobox (ZEB)1, ZEB2, lymphoid enhancer-binding factor 1 and other transcription factors.

The most critical part of EMT is the suppression of E-cadherin synthesis, involved in the formation of tight junctions between epitheliocytes, the increased expression of vimentin, smooth muscle actin, fibronectin and genes, responsible for the mesenchymal phenotype of epitheliocytes. The increased synthesis of extracellular matrix metalloproteinases completes the transformation of precancerous lesions into CRC and distinguishes the cells, capable of invasion into surrounding tissues and penetration into distant organs. In this respect, β-catenin accumulation in the cells of precancerous lesions gives a strong indication that transformation into CRC has been activated (51); however, the existing methods for diagnosing precancerous lesions and CRC, as aforementioned, do not include a standardized immunohistochemical assessment of the nuclear expression of β-catenin, despite the fact that the literature data on chemoprevention of colorectal cancer focuses on reducing the level of β-catenin.

## 6. Inflammatory bowel disease and CRC

Certain difficulties in the differential diagnosis of precancerous lesions and CRC are associated with IBD, involving mucous damage due to infection, allergic reactions or medication, that adversely affects the ability of goblet cells to produce mucin, functioning as a physical and chemical barrier between gut microbiota and mucous membrane (51).

Neutrophils and monocytes are the first to react to the disruption by migrating towards the damaged area with help of chemotactic gradients, formed by interleukin (IL)-1β, IL-6, tumor necrosis factor (TNF)-α cytokines, chemokine (C-C motif) ligand 8, C-X-C motif chemokine ligand 10 chemokines, macrophage inflammatory protein 2, granulocyte-macrophage colony-stimulating factor (GM-CSF), and granulocyte colony-stimulating factor (52).

Neutrophils provide the elimination of pathogens via phagocytosis, the formation of reactive oxygen species and the release of matrix metalloproteinases, elastase and neutrophil extracellular traps. It is generally accepted that once neutrophils complete their task, they should immediately undergo apoptosis, reducing inflammation, promoting the restoration of surrounding tissues and the return to normal tissue homeostasis. Normally, the regulation of local neutrophil activity is performed by immunosuppressive cytokines, in particular, IL-10 and TGF-β, that are produced by M2-activated macrophages, a result of transformation, undergone by monocytes that migrated to the phlogogenic area. In the case of IBD, this does not take place due to excessive phlogogenic stimulation (52).

**Figure 3:**
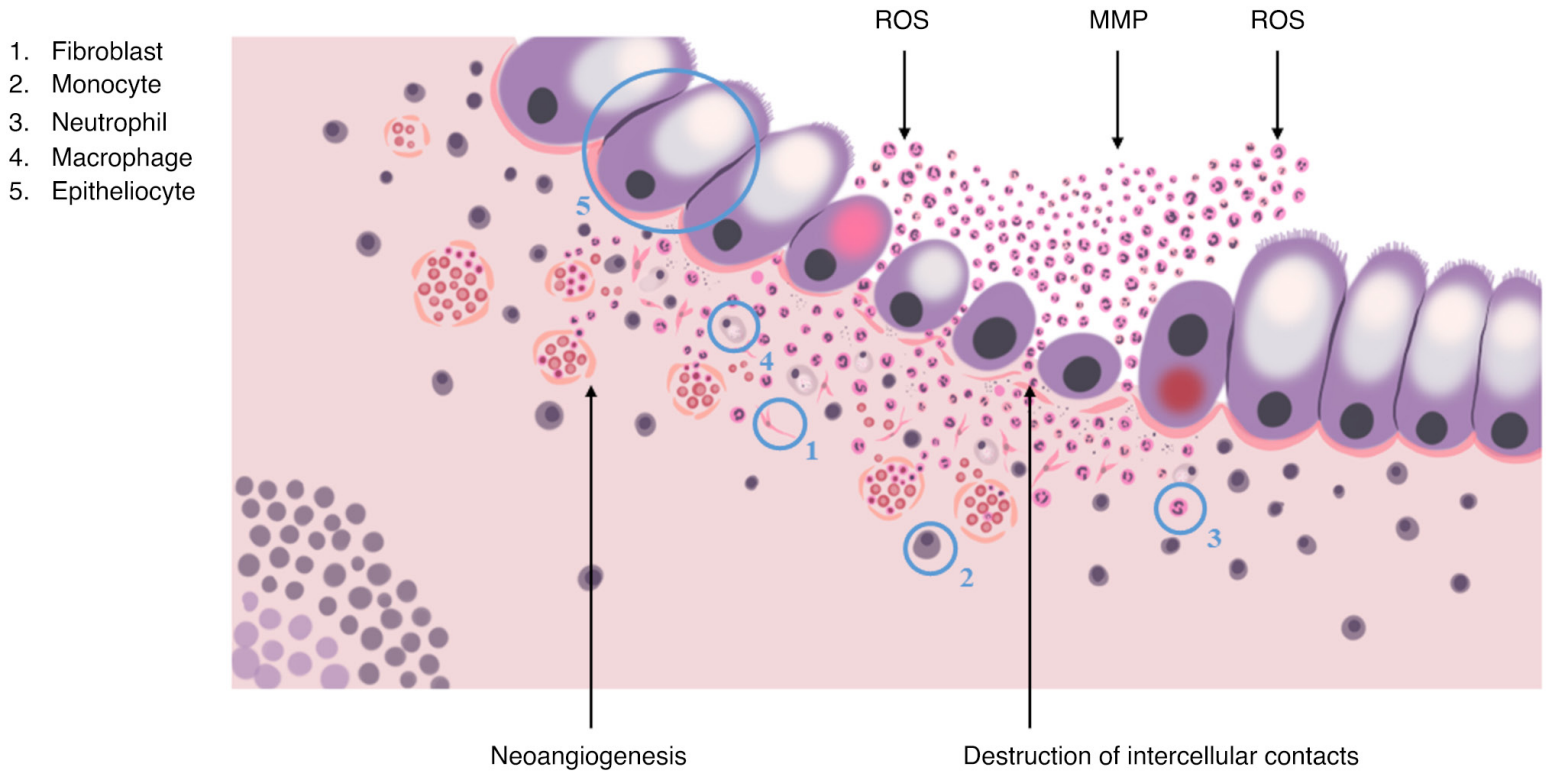
Pathogenesis of the development of cryptitis and crypt abscess. ROS, reactive oxygen species; MMP, matrix metalloproteinase.

The accumulation of activated neutrophils, macrophages and dendritic cells promotes changes in the crypt structure, the disruption of intercellular contacts and integrity of the basal lamina, that leads to the formation of crypt abscesses and is characterized by the increased synthesis of the pro-inflammatory cytokines, TNF-α and IL-1β, and the secretion of non-cytokine inflammatory molecules, attracting T-cells and neutrophils to the area of inflammation (Fig. 3). In turn, M1-activated macrophages in collaboration with intestinal fibroblasts induce fibrotic process, which is a disproportionate synthesis and the accumulation of extracellular matrix components, causing the disruption of mucosal histological architecture, the formation of ulcers and scars of the colonic wall, which significantly complicates endoscopic and morphological diagnosis of CRC (52).

In light of the above, molecular markers indicating the systemic nature of the pathological process are of particular diagnostic value. There is no consensus on such markers, but it is known that neutrophils, monocytes and, to a certain extent, dendritic cells are formed in the bone marrow from a common multipotent progenitor cell of the megakaryocytic lineage that is a direct descendant of a hematopoietic stem cell. It is reasonable to assume that genetic mutations in the stem cell or progenitor cell may prevent immunocytes from performing their regulatory functions, lead to their immortalization and cause disease progression.

A similar scenario was described (53) as one of the possible manifestations of the phenomenon, known as the clonal hematopoiesis of indeterminate potential (CHIP) that is frequently observed during the normal process of ageing (54). The phenomenon is based on the fact that in by the age of 70, hematopoietic stem cells of bone marrow accumulate from 350,000 to 1,400,000 mutations, including *DNMT3A*, Tet methylcytosine dioxygenase 2 (*TET2*), additional sex combs like-1, protein phosphatase 1D, Janus kinase 2 (*JAK2*), splicing factor 3b subunit 1, serine/arginine-rich splicing factor 2, *TP53*, guanine nucleotide binding protein, alpha stimulating activity polypeptide and guanine nucleotide-binding protein. If even one of the mutations appears to be able to guarantee the dominance of corresponding hematopoietic stem cell clone over other clones, a state of clonal expansion appears, which, on the one hand, significantly increases the risk of hematological cancer and, on the other hand, promotes the appearance of mutant immunocyte clones, responsible for a number of chronic inflammatory diseases (54).

Mutations, typical for CHIP, are found in hematopoietic stem cells, monocytes and granulocytes. Individuals with the *DNMT3A* mutation have a high level of IL-6 in their blood; mouse macrophages with *TET2* mutation, when stimulated by bacterial endotoxin, exhibit a significantly increased production of IL-1β, -2, -6 and -8. The *JAK2* mutation is always accompanied by a much stronger activation of granulocytes and the intensified production of IL-6 and -18, without an increase in the C-reactive protein level. Based on this, it is possible to make an accurate assessment of the local processes in the colon, taking into consideration the existing molecular and genetic damage, found in the stem cells and immunocytes (53).

## 7. Chemoprophylaxis

IBD is an unmodifiable risk factor for the development of CRC (54). One of the significant causes of CRC in IBD-associated individuals is chronic inflammation, which is widely acknowledged to promote CRC. The mechanisms responsible for this are not yet fully understood (55). While the suppression of inflammation could potentially lower the risk of IBD-related CRC, there is limited evidence to suggest that anti-inflammatory agents, which are commonly used to treat IBD, have chemopreventive effects on patients with cancer (55).

Decreasing the level of β-catenin, as a key component of the Wnt signaling pathway, plays a crucial role in the prevention of precancerous lesions that are not associated with chronic inflammation. Genes, proteins involved in the signaling pathways of CRC and inhibitors are listed in Table IV (56-58).

### Non-steroidal anti-inflammatory drugs (NSAIDs)

Sulfasalazine and medication with 5-aminosalicylic acid (5-ASA) are considered to be anti-inflammatory preventative drugs. Treatment with sulfasalizine for at least 3 months is considered to have a protective effect, despite the disease activity (59).

A recent meta-analysis confirmed the chemopreventive effects of 5-ASA drugs at a dose of 1.2 g/day in patients with IBD, particularly those with ulcerative colitis, while success was only achieved in prevention of CRC, but not dysplasia (60). Another study demonstrated that mesalamine was associated with risk reduction at the same dosage (61). 5-ASA has chemopreventive effect, enabled via various pathways (62). Induction of S-phase by 5-ASA reduces the frequency of DNA mutations (63,64). Synthase activity and reactive oxygen species formation are also reduced (65,66). The suppression of the EGF and c-Myc pathways induces apoptosis (66,67). The Wnt/β-catenin pathway is modulated and PPAR-γ is activated by 5-ASA, resulting in the inhibition of cell proliferation (68). Finally, 5-ASA inhibits the NF-κB pathway and blocks both the cyclooxygenase (COX)-2-dependent and COX-2 independent growth of cancer cells (69,70). There is evidence to indicate that the inhibitory effect of β-catenin is suppressed (71).

Aspirin is effective for the prevention and treatment of CRC associated with IBD due to inhibition of COX enzymes, since they are the most well-studied targets of aspirin (72). The risk was 20% lower after 5 years of use and 30% lower after 10 years of use, while increasing the dosage up to 100 mg/day decreased the risk by 10%, and up to 325 mg/day, by 35% (73). Aspirin also locally reduces the expression of β-catenin (74).

Presently, to the best of our knowledge, there is no evidence of the protective effect of aspirin against IBD-induced CRC, since patients with IBD exhibit complications due to the induced damage to the colon mucous membrane, with the subsequent development of ulcers, rendering the risk of its use more serious than its potential benefits (75).

Other NSAIDs, such as sulindac, have been proven to induce the apoptosis of colon cancer cells, indicating the class effect of COX inhibitors in the treatment or prevention of CRC that is not related to IBD. The inhibitor of СОХ-2, nimesulide, and the PPARγ ligand, troglitazone, effectively suppress colon carcinogenesis, with nimesulide having a more potent inhibitory effect than troglitazone (76).

Chemoprophylactic medication for non-IBD-related CRC also includes aminosalicylates due to their ability to inhibit COX, lipoxygenases, platelet-activating factor, IL-1β and eliminate reactive oxygen species (77).

According to scientific publications, the use of 400 mg Celecoxib daily reduces the incidence of adenoma relapses by 34%, lowers the risk of advanced adenomas development by 55% and provides a 7-fold lower chance of developing fatal outcomes (78).

### Antiparasitic drugs

Bezafibrate also reduces the incidence of colorectal adenocarcinoma, although to a lesser extent than nimesulide and troglitazone. In colorectal adenocarcinoma, the use of nimesulide, troglitazone and bezafibrate has been shown to suppress cell proliferation activity, induce apoptosis, and decrease β-catenin, COX-2, inducible nitric oxide synthase and nitrotyrosine immunoreactivity (76). Niclosamide is also able to reduce β-catenin expression by disrupting the metastasis-associated in colon cancer 1-β-catenin-S100A4 axis (79).

### Probiotic bacteria

The use of probiotic bacteria is a novel method used for the prevention of carcinogenic exposure. Conjugated linoleic acid production in mouse models of CRC activates PPAR-γ, which inhibits COX-2 and induces apoptosis (80).

### Glucocorticosteroids (GCs)

GCs, which include endogenous substances, such as cortisol, cortisone and corticosterone, are secreted by the hypothalamic-pituitary-adrenal (HPA) axis, which is affected by various factors, such as circadian rhythm, stress and inflammatory stimuli. When IL-1, TNF and IL-6 activate the HPA axis, the production of GCs and the secretion of GCs is stimulated (78).

Inflammation is indirectly regulated by the HPA axis, which inhibits the activation, migration and proliferation of immune cells of both the innate and adaptive immune systems, mediated by the glucocorticoid receptor (GR) located in the cytoplasm. The glucocorticoid receptor is associated with heat shock proteins, immunophilins, kinases and phospholipases (receptorosomes), which form a complex. Following spatial changes and GC/GR interaction, GR dissociates from receptorosomes and translocates to the nucleus (78).

An enzyme known as activated GR exerts a genomic effect caused by a DNA-binding sequence that contains two zinc finger motifs. Specific glucocorticoid response elements are at the core of this sequence, which affect various genes, including proinflammatory mediators and transcription factors (e.g., activator protein-1 and NF-κB). At the same time, GC/GR trigger an increase in the expression of IL-1 receptor antagonists, Iκ-B and lipocortin-1. These transcriptional effects are responsible for the immunoregulatory and anti-inflammatory effects of GR. Glucocorticoid-induced leucine zipper is a significant target of GC/GR transcriptional activity, as it affects the mitogen-activated protein kinase pathway and NF-κB transcriptional activity, ultimately leading to the regulation of immune-mediated and inflammatory responses (78). Budesonide and other GCs are associated with the suppression of the function of the HPA axis and endogenous cortisol levels. The immunosuppressive effects of corticosteroids (budesonide, hydrocortisone, prednisolone and methylprednisolone) are reduced by decreasing the pathological production of IL-1, -2, -3, -4, -5, -6, -8, -10 and -12, and TNF-α, interferon-γ and GM-CSF (81).

The reduced synthesis of anti-inflammatory cytokines leads to remission in patients with active IBD. However, the medications are not recommended for long-term use due to severe side-effects (82), including peptic ulcers, cataracts, hypertension, adrenal atrophy, amenorrhea, type II diabetes, hyperglycemia, Cushing's syndrome, a high risk of infection, osteoporosis and avascular necrosis. According to the study by Lichtenstein *et al* (83), the use of steroids is associated with both progression and a risk of mortality in patients with IBD, when compared to immunomodulators and biological therapies. Between 1994 and 2008, Targownik *et al* (84) discovered that steroids only had an effective efficacy of 50% in patients with IBD within the first 5 years of diagnosis, which increased to 62% in the initial 10 years, despite progress in biotherapy.

### Immunomodulators

Immunomodulators can be used to achieve lasting remission and are commonly prescribed in patients who are not responsive to aminosalicylates and corticosteroids, or as a supplement to anti-TNF therapy for antibody prevention, particularly with infliximab (85,86).

### Biological therapy

Bioengineered antibodies target specific molecules or proteins that cause inflammation or participate in inflammatory process; among these, there is an adhesion molecule antagonist (vedolizumab, natalizumab), a drug targeting IL-23/IL-12 (ustekinumab) (87). The effects of ustekinumab treatment may be determined by the modulation of IL-23 expression and the levels of miR-29. For example, for miR-126 and vedolizumab, the potential is the same; Harris *et al* (88) explained how endogenous miR-126 inhibits leukocyte adhesion through the regulation of vascular cell adhesion molecule-1, an adhesion molecule expressed by endothelial cells.

### TNF-α inhibitors

During bowel inflammation, TNF is produced by different immune cells, including macrophages, T-cells and dendritic cells, in the intestine of patients with IBD (89), to induce neoangiogenesis (90). In addition, different immune cells of mucous membrane are activated to produce pro-inflammatory cytokines and stimulate the death of Paneth cells via necroptosis (91) or to induce the apoptosis of colon epithelial cells (92). Therefore, inhibiting TNF can suppress colon inflammation. Anti-TNF drugs induce and sustain the healing of mucous membranes in cases of moderate and severe IBD and, as a result, may have chemoprophylactic advantages by reducing long-term chronic inflammation (93). TNF-α has been reported to promote inflammation and IBD-CRC, facilitating DNA damage, stimulating angiogenesis and inducing COX-2 expression that also induces angiogenesis, resulting in tumor growth. TNF-α expression, as demonstrated using mouse models, is associated with development of colon cancer, while the inhibition of TNF-α reduces inflammation and tumor growth; the effect is particularly especially visible in mice, having received the anti-TNF agents, infliximab and etanercept (94,95). Modern studies have not proven that TNF inhibitors prevent dysplasia development (93).

Some studies have shown that tofacitinib inhibits JAK-1, JAK-2 and JAK-3, thus blocking the signaling pathways of cytokines, containing γ-chain, mostly IL-2, IL-4, IL-7, IL-9, IL-3, IL-5 and IL-21. Notably, JAK inhibition has been found to be effective in suppressing T-cells, natural killer cells and modulating pro-inflammatory cytokines; this opens up the possibility of simultaneously blocking the activity of several pro-inflammatory cytokines (96).

### Thiopurines

Thiopurines have been used to maintain remission in patients with IBD in order to avoid a long-term use of steroids (97). However, a connection has been reported between the use of thiopurines and a higher risk of developing lymphoproliferative malignant neoplasms in 5% of cases (98). A previous meta-analysis revealed an association between treatment with thiopurine and the risk of CRC development in patients with IBD, particularly the ones with ulcerative colitis (99). Some data have been published, indicating the reduction of a high degree dysplasia and CRC both in case-control studies, and cohort ones (78). A more significant chemoprophylactic effect was recorded in patients who are at a high risk of developing CRC, having the disease for >8 years. However, this study did not discover any protective effect in patients with IBD or extensive colitis (99). Although thiopurines are known to decrease the risk of developing CRC in patients with IBD, they may have carcinogenic properties. As a result, the 2017 European Crohn's and Colitis Organisation (ECCO) consensus did not recommend chemoprophylaxis with thiopurines (100).

### Statins

Specifically, statins target the 3-hydroxy-3-methyl-glutaryl-coenzyme A reductase (HMG-CoA reductase) enzyme, which in turn reduces cholesterol production and promotes the elimination of low-density lipoproteins. A number of patients with hyperlipidemia use these drugs. For prophylaxis and the treatment of CRC, the use of statins is also critical (101). Researchers have indicated that statins can exert a chemoprophylactic effect on CRC by targeting the inflammation-induced proliferation of colon cancer and potentially affecting intracellular oxidative stress, apoptosis and vascular endothelial growth factor inhibitors (102). However, studies on the impact that statins have on CRC in patients with IBD are limited; therefore, the chemoprophylactic use of statins for patients with IBD remains a controversial topic (103-106).

### Ursodeoxycholic acid

Patients with IBD and primary sclerosing cholangitis (PSC) have a 5-9-fold higher risk of CRC than patients with IBD (107,108). High level of bile acids in the colon may produce carcinogenic effects, leading to the proliferation of colonic epithelial cells, ultimately leading to the development of dysplasia or CRC (109,110). Researchers have suggested that ursodeoxycholic acid may reduce colonic dysplasia in patients with ulcerative colitis and PSC (110), although these data are controversial (109).

### Folic acid

Patients with IBD may suffer from foliate deficiency due to inadequate nutrition, the competitive inhibition of intestinal absorption of sulfasalazine and excessive luminal losses (111,112). Chemoprophylaxis with folic acid is feasible due to its low cost, good tolerability and safety. Further studies are required however, to determine the chemopreventive effects of folic acid.

## 8. Conclusion and future perspectives

Despite the advances of modern pharmacological industry, the chemoprophylaxis of CRC for patients with precancerous lesions remains rather ineffective. Precancerous colorectal lesions are detected daily via endoscopic methods and are confirmed pathomorphologically. Patients with IBD are also left to constantly balance between the remission and exacerbation stages. The key to success may be the local reduction of nuclear β-catenin that is the main initiator of oncogenic transformation of precancerous lesions into CRC. The immunohistochemical evaluation of nuclear β-catenin hyperexpression in precancerous cells may aid in the differential diagnosis of early-stage cancer and dysplasia, and may improve the assessment of the treatment efficacy in IBD itself and IBD-associated lesions.

Multiple data on the effectiveness of chemoprevention remain conflicting; there are no reliable data on the reduction of nuclear β-catenin expression in studies on most pharmacological drugs, and in some cases, the supposed benefit is overshadowed by existing side-effects, particularly as regards GCs and thiopurines. There is also an issue with the cross-inhibition of drugs. Therefore, further studies are warranted to investigate the phenomenon of clonal hematopoiesis with uncertain potential, which is probably a decisive factor in the progression of IBD, and further studies of repurposed drugs are also required.

## Figures and Tables

**Table I:** The most common genetic hereditary syndromes of colorectal cancer.

Name of hereditary syndrome	Type of inheritance	(Refs.)
Familial adenomatous polyposis (FAP) syndrome	Autosomal-dominant	(113)
Mutated MMR gene syndrome	Autosomal-dominant	(114)
Lynch syndrome	Autosomal-dominant	(115)
Muir-Torre syndrome	Autosomal-dominant	(116)
Peutz-Jeghers syndrome	Autosomal-dominant	(117)
Cowden syndrome	Autosomal-dominant	(118)
Juvenile polyposis syndrome	Autosomal-dominant	(119)
Serrated polyposis syndrome (SPS)	Unknown	(120)
Cronkhite-Canada syndrome	No reliable data available	

MMR, DNA mismatch repair.

**Table II:** Common lifestyle factors of colorectal cancer.

Risk factors	(Refs.)
Low socioeconomic status	(121,122)
Overweight and obesity	
Sedentary lifestyle	
Smoking tobacco	
Alcohol abuse	
Low fiber, high fat diet	
Consumption of red and overcooked meat	
Insulin-resistant diabetes mellitus	
Acromegaly	
Organ transplantation with long-term immunosuppression	
Long-term androgen deprivation	

**Table III:** Precancerous lesions and subtypes of the colon.

Name of lesions	Types	(Refs.)
Polypoid lesion with serrated histoarchitecture	HP/hyperplastic polyp	(18)
	MVHP/microvesicular hyperplastic polyp	
	GCHP/goblet-cell-rich hyperplastic polyp	
	SSL/sessile serrated lesion with dysplasia or not	
	TSA/traditional serrated adenoma	
Tubulovillous adenomas	TA/tubular/conventional adenoma	
	TVA/villous/tubulovillous adenoma	
Focal intraepithelial neoplasia in chronic inflammatory bowel disease	Inflammatory bowel isease	

**Table IV:** Genes, proteins and inhibitors.

Genes	Proteins	Pathways in colon cancer	Inhibitors	Source/(Refs.)
*KRAS*	KRAS	ERK, PI3K/AKT/mTOR, RAS, regulating pluripotency of stem cells, WNT signaling pathways	Glecirasib	MedChemExpress
			Rineterkib	
			Sotorasib	
			Adagrasib	
*BRAF*	BRAF	ERK, PI3K/AKT/mTOR, RAS, WNT signaling pathways	Uplarafenib	MedChemExpress
			Lifirafenib	
			Tinlorafenib	
			Anticancer agent 124	
			Exarafenib	
			Avutometinib	
			Belvarafenib	
*APC*	APC/DP2.5	WNT signaling pathways	4-APC hydrobromide	MedChemExpress
			Eftilagimod alfa	
			Drotrecogin alfa activated	
			Danicopan	
*TP53*	p53/TRP53	Mutation-inactivated TP53 to transcription	Kevetrin hydrochloride	MedChemExpress
			PRIMA-1	
			Eprenetapopt	
			Rebemadlin	
*CTNNB1*	Catenin beta-1/β- catenin	PI3K/AKT/mTOR, WNT signaling pathways	SSTC3	MedChemExpress
			5-aminosalicylic acid	
			Aspirin	
			Nimesulide	
			Troglitazone	
			Bezafibrate	
*PIK3CA*	P110α	PI3K/AKT.mTOR, ERK, TNF, PD-L1 expression and PD-1 checkpoint pathways in cancer	Alpelisib	MedChemExpress
			Vevorisertib	
			Taselisib	
			Vevorisertib	
			Trihydrochloride	
*C-MYC*	bHLH	Wnt, JAK-STAT signaling pathways	Idarubicin	MedChemExpress
			hydrochloride	
			Agrimol B	
			Mollugin	
*CYCD*	CDKs	Wnt, Hippo, JAK-STAT signaling pathways	Alvocidib	MedChemExpress
			Seliciclib	
			Palbociclib	
			Ribociclib	
			Abemaciclib	
*WISP1*	WISP1	Wnt signaling pathways	Cabazitaxel	MedChemExpress
			Docetaxel	
			Paclitaxel	
*JAK2*	JAK2	PD-L1 expression and PD-1 checkpoint, JAK-STAT, PI3K/AKT/mTOR signaling pathways	Tofacitinib	MedChemExpress
			Baricitinib	
			Ruxolitinib	
			Upadacitinib	
			Fedratinib	
			Abrocitinib	
*GNB1*	GNB1	PI3K/AKT/mTOR, RAS, MAPK signaling pathways	BEZ235	(56)

*KRAS*, Ki-ras2/Kirsten rat sarcoma viral oncogene homolog; *BRAF*, V-Raf/murine sarcoma viral oncogene homolog B; *APC*, adenomatous polyposis coli; *TP53*, tumor protein p53; *TP53*, tumor protein p53; *CTNNB1*, catenin beta-1; *PIK3CA*, phosphatidylinositol-4,5-bisphosphate 3-kinase catalytic subunit alpha; *C-MYC*, cellular myelocytomatosis oncogene; *CYCD*, cyclin-dependent kinase 4; *WISP1*, WNT1-inducible signaling pathway protein-1; *JAK2*, Janus kinase 2; *GNB1*, guanine nucleotide-binding protein; ERK, extracellular-regulated kinase; PI3K, phosphatidylinositol-3-kinase; AKT, protein kinase B; mTOR, mammalian target of rapamycin; TNF, tumor necrosis factor; PD-L1, programmed death-ligand 1; STAT, signal transducer and activator of transcription; MAPK, mitogen-activated protein kinase.

## Data Availability

Not applicable.
